# The implementation of a physical activity intervention in adults with Obstructive Sleep Apnoea over the age of 50 years: a feasibility uncontrolled clinical trial

**DOI:** 10.1186/s13102-020-00195-8

**Published:** 2020-08-08

**Authors:** Julie K. Black, Anna C. Whittaker, Abd A. Tahrani, George M. Balanos

**Affiliations:** 1 School of Sport, Exercise and Rehabilitation Sciences, College of Life and Environmental Sciences, University of Birmingham, Edgbaston, Birmingham, West Midlands B15 2TT UK; 2 Faculty of Health Sciences and Sport, University of Stirling, Stirling, Scotland, FK9 4LA UK; 3 Institute of Metabolism and Systems Research (IMSR), The Medical School, University of Birmingham, Birmingham, UK; 4 Centre of Endocrinology, Diabetes and Metabolism (CEDAM), Birmingham Health Partners, Birmingham, UK; 5 Department of Endocrinology, University Hospitals Birmingham NHS Foundation Trust, Birmingham, UK

**Keywords:** Physical activity, Obstructive sleep apnoea, Cardiovascular disease, Sleep disorders, Ageing

## Abstract

**Background:**

Obstructive Sleep Apnoea (OSA) is a risk factor for cardiovascular disease (CVD) and Type 2 diabetes (T2D). Observational studies suggested that OSA treatment might reduce CVD and T2D but RCTs failed to support these observations in part due to poor adherence to continuous positive airway pressure (CPAP). Physical activity (PA) has been shown to have favourable impact on CVD and the risk of T2D independent of its impact on weight and therefore might provide additional health gains to patients with OSA, whether or not adherent to CPAP.

**Methods:**

The main aim of this study was to explore the feasibility of providing a 12-week PA intervention to adults aged over 50 with OSA. The secondary aim was to assess the impact of the PA intervention on OSA severity. Patients with moderate-severe OSA (apnoea hypopnea index (AHI) ≥ 15 events/hour (based on overnight ApneaLink™) were recruited in response to posters displayed in workplaces. A 12-week daily PA intervention was delivered in participant’s home setting and PA was monitored via text and validated by objective PA measures (GT3X accelerometers).

**Results:**

The intervention was feasible as all 10 patients (8 males, mean (SD) age 57.3 (6.01)) completed the intervention and PA increased across the 12-weeks. The duration of PA increased from baseline (113.1 min (64.69) per week to study-end following the intervention (248.4 min (148.31) (*p* = 0.02). Perceived Exertion (RPE) (physical effort) increased significantly between baseline (M = 10.7 (1.94)) to end of intervention (M = 13.8, (1.56) (*p* < 0.001). The intervention had no significant impact on weight or composition. Following the intervention, there was a statistically non-significant a reduction in AHI from baseline to study end (22.3 (7.35) vs. 15.8 (7.48); *p* = 0.09).

**Conclusion:**

It is feasible to deliver a PA intervention to adults aged over 50 with OSA. The intervention resulted in improved PA and AHI levels somewhat and seemingly independent of weight changes. Future trials need to examine whether PA can reduce the burden of OSA associated comorbidities.

**Trial registration:**

CTN: ISRCTN11016312 Retrospectively Registered 21/07/20.

## Background

Obstructive Sleep Apnoea (OSA) is a common disorder that is currently estimated to affect approximately one billion people worldwide [1]; with high prevalence in many countries such as the U.S. (34% men and 17% women), and Switzerland (50% men and 23% women) among others [1]. OSA prevalence increases with age until up to 60 years old [2], hence, many middle-aged men and women are at increased risk of developing OSA [3–5]. OSA is characterized by instability in the upper airways (UAs) during sleep leading to recurrent episodes of the UA obstruction [3]. These repeated obstructions are associated with recurrent episodes of oxygen desaturation/reoxygenation, cyclical changes in blood pressure (BP), and heart rate, and increased sympathetic activity and intrathoracic pressure, brief microarousals and changes to sleep architecture, such as the loss of REM and slow wave sleep [6]. OSA is associated with several comorbidities including increased risk of cardiovascular disease (CVD) [7], Type-2 Diabetes (T2D) and its microvascular complications [8–11], and hypertension [12], and thus, has serious implications for health and wellbeing across the lifespan.

Currently, the gold standard treatment for OSA is continuous positive airway pressure (CPAP) [13]. Recently, it has been suggested that PA could be used as an adjunctive to CPAP [14]. Observational studies have shown the potential benefits of CPAP, which include protection from heart failure (HF), T2D and CVD. However, interventional studies have demonstrated that CPAP, either with or without lifestyle advice, does not protect against CVD [15] Thus, the potential clinical benefits of CPAP use in this respect are questionable and that PA may be a viable management tool for reducing the risk of CVD and the severity of OSA. Additionally, overall adherence to CPAP is often poor due to discomfort with the mask and the general intrusion of the machine during sleep time amongst other factors [16–18].

Although obesity is the main risk factor for OSA [19], the lack of regular physical activity (PA) (30 min of moderate to vigorous intensity, 5 days per week) also increased the risk of OSA development [20–22]. As such, it is plausible that PA might play an important role in the pathogenesis and management of patients with OSA. In addition to the potential direct benefits of PA on OSA; PA and active lifestyle have been shown to reduce CVD and T2D, independent of weight loss [23], although whether this remains true in patients with OSA remains unknown. Furthermore, cardiorespiratory fitness has been shown to independently determine health status [24].

We hypothesized that PA can improve cardiorespiratory fitness and improve OSA severity possibly via facilitating upper airway adaption and rostral fluid shift [25, 26]. Hence we conducted a study to assess the feasibility of conducting a 12-week PA intervention in adults aged 50 years and above with OSA. The secondary aim was to assess the impact of the PA intervention on the severity of OSA based on AHI).

## Methods

### Aim, design and setting

An uncontrolled feasibility single arm trial to deliver a 12-week PA intervention to a group of adults aged 50 years and over in participants’ home setting.

### Participants

Participants were recruited via poster placed in workplaces and around local villages by the principal investigator. The aim was to recruit 30 participants [27] as this was a feasibility trial, so no formal power calculation was made.

Inclusion criteria: 1. Adult manual workers (e.g. construction, prison service) an underserved population in this area of research. Older manual workers are known to have low levels of leisure time physical activity and OSA might impact longevity in the workplace, who were employed for at least three months before the start of the study, 2. had no previous diagnosis or treatment for obstructive sleep apnoea (OSA) and were willing to be screened for OSA and to participate in a 12-week physical activity (PA) intervention were eligible for the study. The diagnosis of OSA was made using an ApneaLink™ device for one night and only those with AHI > 15 events/hour were invited to participate in the study [28]. Exclusion criteria included those younger than 50 or older than 69 years old, unemployed or in employment less than three months at commencement of study, had history of chest pain during exercise or heart trouble, or with a diagnosis of and currently undergoing treatment for OSA. The University of Birmingham Science, Technology, Engineering and Mathematics ethics committee approved the study (ERN_16–1326). All study participants signed informed consent before enrollment to the study.

### Measures

#### Subjective physical activity

The short form International Physical Activity Questionnaire (IPAQ) was used to measure habitual levels of physical activity over the last seven days. The IPAQ short form has been tested in 12 countries and has been shown to produce good repeatable (75%) and reliable data in middle aged employed samples [29] with median internal consistencies of α = .80. Concurrent validity and criterion validity have been supported; the scale can correctly classify low, moderate and high intensity activity [29]. The Cronbach’s alpha for internal consistency in the present study was α = .77.

#### Objective physical activity

GT3X accelerometers (ActiGraph, Pensacola, FL, USA) were used to validate subjective reports of PA and intensity. The GTX3 has demonstrated high intra and inter-instrument reliability with an intra-class correlation coefficient for activity counts of 0.97 [30]. The accelerometer was worn as a belt on the right hip and collected motion data as suggested by [31]. Activity counts were produced as a result of the summed acceleration measured in the duration of collection over seven days and represented a measure of activity over time. Cut-points of < 1951, 1952–5724, > 5725 for light, moderate and vigorous intensity respectively, were used to determine levels of low, moderate and vigorous intensity physical activity [31, 32].

#### Increase in daily physical activity

Borg’s Rating of Perceived Exertion (RPE) scale was used to assess physical activity intensity as perceived by participants. Borg’s RPE is an affordable and reliable method of measuring subjective perceived effort during exercise and has been demonstrated as a valid measure of intensity relating to heart rate [33].

#### Anthropometric measures

Height and weight were measured before the intervention commenced to calculate body mass index (BMI) (kg/m^2^). Electronic scales with bioelectrical impedance analysis (Salter Ultra Slim Glass Analyser scales, model 9141, HoMedics Group Ltd., Kent, UK) were used to measure weight and body fat percentage. Bioelectrical impedance analysis has been shown to be a valid and cost-effective measure of body composition [34]. A Bosch PLR 30c Laser Measure (Robert Bosch GmbH, Germany) was used to measure height.

#### Obstructive sleep Apnoea (OSA)

Participants were assessed for OSA using an ApneaLink™ device. The device has been shown to be a reliable and valid method of detecting the presence and risk of OSA in patients aged > 50 years [35]. It has a sensitivity for AHI > 10 of 92–100% and a specificity of 87–96.7% [28, 35, 36]. OSA presence and severity was based on AHI (normal < 5, mild 5- < 15; moderate 15- < 30 and severe > = 30) [4, 37, 38]. Apnoea was defined as a reduction in flow to less than 90% of normal and hypopnea as a reduction in flow to 30% of normal with 4% desaturation for at least 10 s [39].

#### Feasibility and retention

Feasibility was assessed in relation to participant compliance and adherence to the intervention. Compliance and adherence to the intervention of at least 80% was deemed as feasible. Participant retention was also deemed successful at 90%.

#### Study procedures

Following consent, eligible participants with an AHI score greater than 15 were invited to take part in the intervention. Baseline data collected included: information regarding the participant general health, anthropometric measures, and current daily physical activity levels (IPAQ).

At baseline, participants were advised in a 15-min face to face session by the principal investigator, as to how to increase the frequency, duration and intensity of their current PA levels (e.g. taking the stairs instead of the lift) over the 12 weeks (Table 1). RPE was used to gauge appropriate % increase in intensity ranging from a score of 9 to a score of 16. An increase in exercise intensity from 9 to 11 on the Borg scale was deemed suitable for less trained; this was advised at the start of the intervention and then increased upwards as required [33, 40]. Participants were asked to report their total daily amount of PA and intensity (RPE) as two numbers (minutes/duration of PA and number corresponding to RPE on the Borg scale) to the mobile phone of a researcher via text message. Levels of PA were monitored by GTX3 accelerometers at three time points for 7 days (during weeks 1, 6, and 12). Participants were asked to wear the accelerometer on a belt on the right hip from the moment they got up until they went to bed. Removal of the accelerometer was instructed for showering, bathing and swimming. Total minimum wear time required was 12 h for seven days [31]. At the end of each seven-day period a researcher collected and downloaded the recorded data from the accelerometer. The accelerometer data was then compared to the self-report PA scores. At the end of the 12-week period, post intervention measurements including AHI were taken as described earlier and compared to those at baseline to assess effectiveness of the intervention.

**Table 1:** Structure of 12-Week Physical Activity Intervention

Weeks	Duration (Mins)	RPE	Frequency (days per week)
1–4	10–15	9	3
5–8	15–20	9–13	4
9–12	30	13	5

*RPE* Rate of Perceived Exertion

#### Data analysis

Data analysis was performed using IBM SPSS Statistics software version 25. Data are presented as mean difference scores (95% CI) and effect sizes. In addition, for informative purposes, paired samples t-tests were performed to assess change of PA levels over time in minutes and perceived exertion (RPE) from beginning to end of intervention. The same tests were also used to investigate change over time in AHI (OSA severity), PA, weight and fat pre- to post-intervention. Where the assumption of normality was violated for any variable, the non-parametric Wilcoxon signed-rank test was used instead. Pearson correlations were used to assess relationships between continuous variables on the change scores from pre-post intervention. Significance was assumed where *p* < 0.05.

## Results

### Intervention selection

**Fig. 1:**
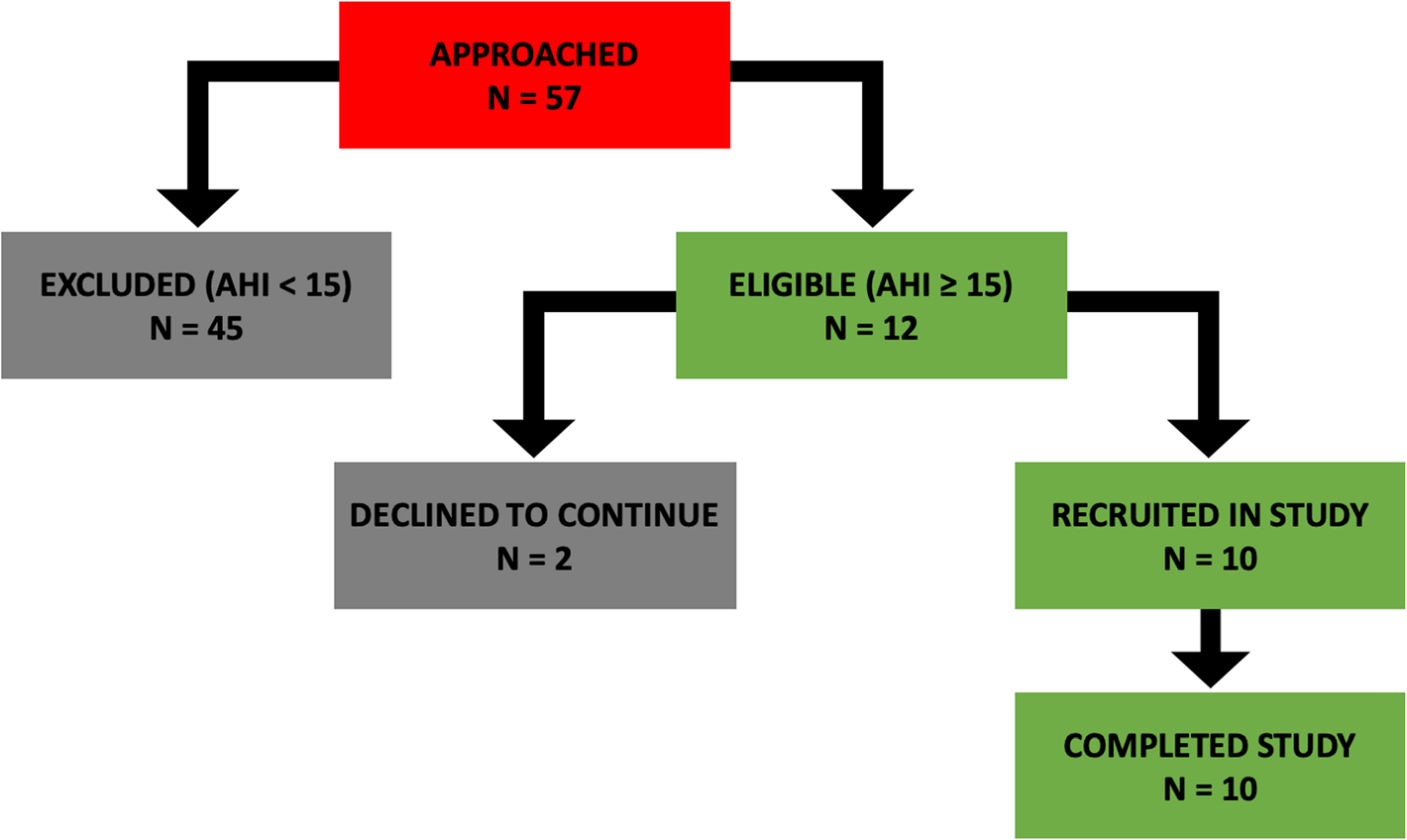
Flow chart showing process of screening and recruitment

A total of 59 participants who responded to displayed posters were screened for inclusion in the intervention study. Of the original pool of participants, 49 were excluded due to an AHI score of less than 15. Ten eligible participants (mean age 57.3 (6.02) years, 8 males), with an AHI score greater than 15, were recruited in the intervention. A flow diagram of screening, recruitment and intervention completion is illustrated in Fig. 1 below.

Baseline characteristics for all participants are shown in Table 2. All 10 participants who were recruited in the study completed the intervention, there were no drop outs, although some missing data was present for certain variables. Participants were middle-aged men and women with moderate OSA who were non-smokers, were overweight or obese and were mostly sedentary. Their alcohol intake was less than 14 units per week.

**Table 2:** Descriptive statistics of the sample (*n* = 10) at baseline. Data presented as mean (SD)/Number (*N*)

	***N***	Minimum	Maximum	Mean	Std. Deviation
BMI	10	25.00	46.00	32.9000	7.23341
Weight -Base (Kg)	10	76.00	130.00	96.6000	17.69620
Body Fat - Base	10	31.00	79.00	46.1000	15.28580
AHI - Base	10	15.00	39.00	22.6000	6.97933
ActivityMinsWk1	10	30.00	220.00	107.7000	62.60112
ActivityRPE1	10	9.00	14.00	10.5000	1.90029
Age	10	50.00	69.00	57.3000	6.01941
IPAQ – Base	10	1.00	3.00	2.0000	.47140
Valid N (listwise)	10				

### Primary aim analysis

All 10 participants (100%) complied with and adhered fully to the 12-week intervention thus, the feasibility of delivering the intervention was confirmed, although data was missing (two participants without fat % measurements and 1 each for RPE, AHI and weight).

### OSA and physical activity levels

Lower levels of baseline self-reported PA (IPAQ) were associated with higher baseline AHI score.

### The impact of the intervention

The impact of the intervention is summarized in Table 3. Analysis revealed that compared to baseline, after the intervention there were significant improvements in PA duration and RPE, and a trend towards an improvement in AHI without any changes in body weight or body composition. The effect sizes for the change in PA and RPE are considered large, and for AHI the effect was medium. Time spent in PA was confirmed by accelerometer in week one with an average count of 1515, indicating light PA, and in week 12 with an average count of 2060, indicating moderate PA (Freedson et al. 1998).

**Table 3:** Changes in variables from baseline to end of intervention. Data presented as mean (SD)

Variable	Baseline	End	Mean Difference	95% CI	Hedges	*P* value	*N*	Hedges’g (Effect size)
PA min	113.1 (64.69)	248.4 (148.31)	− 135.25	− 251.7 to −18.8	−0.85	0.02	8	−1.115
RPE (effort)	10.7 (1.94)	13.8 (1.56)	−3.11	−4.4 to −1.9	−1.71	0.001	9	−1.660
AHI	22.3 (7.35)	15.8 (7.48)	6.56	−1.2 to 14.3	0.56	0.09	9	0.826
Weight (kg)	98.6 (17.59)	98.7 (15.30)	−0.11	−2.1 to 1.9	− 0.04	0.90	9	−0.006
Fat (%)	43.4 (10.94)	43.0 (11.56)	0.38	−3.5 to 4.2	0.07	0.67	8	0.034

Correlations between AHI change scores (pre-post) and BMI, weight, and fat show that the trend for a reduction in AHI was seemingly independent of the change in BMI (*r* = −.51, *p* = .16), weight (*r* = −.34, *p* = .36), and fat (*r* = .11, *p* = .80).

## Discussion

Our study demonstrated that it is feasible to deliver a 12-week PA intervention to patients with OSA aged above 50 years old in a community setting, and that a structured PA program might have the potential to improve PA and OSA severity independent of weight loss.

Currently, the debate relating to the mechanisms by which PA reduces OSA severity is unresolved. Although previous research has demonstrated that increased ventilator muscle tone may be key [26], others disagree [41]. Additionally, Redolfi et al., [25] demonstrated that a decrease in fluid shift towards the neck during sleep as a result of walking increased the space in the pharyngeal region. Although, each of the above suggestions is logical, more recent evidence suggests that OSA reduction might be as a result of reduced inflammation and improved autonomic function via exercise, [41–43]. These in turn, might potentially improve chemosensitivity and upper airway stability.

The present study gives tentative support for a PA intervention to reduce OSA. In contrast to previous research that utilized supervised interventions, the PA intervention in the present study was unsupervised [43–45]. As such, the potential of unsupervised exercise to reduce OSA might be advantageous on two counts. Firstly, to those who find it difficult to fit scheduled exercise into their day, and secondly promotes greater frequency of movement over a 24-h period [46]. The present study also demonstrates the feasibility of delivering a PA intervention to older manual workers who are an underserved population in this area of research. Indeed, older manual workers have been previously shown to have low levels of leisure time PA, thus, together with their increasing age, older workers are likely candidates for OSA development [47–49]. Previous research into exercise as an intervention for OSA management, has largely used structured/planned supervised exercise, and although indications are positive in this effect, it is unknown as to whether participants are likely to adhere to such structure beyond intervention end [41, 45, 50, 51]. In this respect, the present study demonstrated that it is possible for a group of OSA sufferers aged 50 and over to adhere to a PA intervention, however, in order to assess whether the nature of the intervention would be sustainable in the long-term requires further research. Thus, more longitudinal studies are necessary in this respect to determine this outcome. Additionally, findings from the present study demonstrate the potential of daily-unstructured PA to somewhat reduce OSA severity. This is encouraging, since daily general PA such as walking or using the stairs instead of lifts, may be easier to incorporate into daily life and therefore, more sustainable in the long-term, as opposed to structured exercise which requires planning. Indeed, Chaput, Carson [46] suggests that PA spread over a 24-h period may be more beneficial to health than single bouts of planned exercise.

The present study also offers support to the recommendations of daily levels of PA to preserve health and prevent disease and demonstrates that the recommended daily activity levels for adults may be sufficient to somewhat reduce or potentially prevent OSA and its development. Further, the contribution of regular daily PA, may offer an alternative means of OSA management, especially for those who find they cannot tolerate treatments such as CPAP and also help to offset the risk of CVD in OSA sufferers [52, 53]. Although the exact mechanism by which cardiorespiratory fitness decreases the risk of CVD is unclear, possible mechanisms such as an improvement in arterial function and anti-inflammatory effects have been suggested [54]. It has also previously been suggested that regular PA may improve inflammatory profiles associated with OSA and CVD such as activation of the peripheral nervous system and others that predispose an individual to vascular damage [42]. Although CPAP is still the recommended gold standard treatment for OSA management, it does not promote the participation of regular physical activity, which has far reaching benefits for health and wellbeing [55].

### Limitations and strengths

It must be noted that the present study is not without limitations. Firstly, the sample size was very small and thus, findings from the present study cannot be generalized to other groups. Additionally, the sample was composed mainly of Male vs Female and thus is a possible biased that must be acknowledged. However, the present study is a feasibility study and thus, may serve as a platform to guide similar interventions in this age group. Secondly, the daily PA duration and intensity across the 12 weeks were self–reported and so may be prone to recall bias and over-reporting. However, the PA levels were validated at three time points across the intervention with accelerometers and as such validate the reported levels of PA. The present study demonstrated a significant increase in activity levels and a trend towards a change in OSA severity post-intervention which seems independent of the change in BMI and weight-loss. Further, the changes demonstrated in OSA severity (which cannot be manipulated or influenced) suggest that intervention PA adherence was good.

The strengths of the present study should also be noted. To our knowledge, this is the first feasibility study into this group of participants that has used unstructured general PA to reduce OSA. Additionally, all of the participants were older workers, aged 50 years and over who were not undergoing any treatment for OSA.

## Conclusions

A 12-week PA intervention using general daily PA can be delivered and adhered to adults aged 50 years and over with OSA. PA reduced OSA severity somewhat and independent of weight-loss and BMI. This suggests that incorporating more general activity during daily life may prove a feasible route to the management of OSA, and as such warrants examination in full RCT.

## Data Availability

The datasets used and/or analysed during the current study are available from the corresponding author on reasonable request.

## Abbreviations

OSA: Obstructive Sleep Apnoea
T2D: Type 2 Diabetes
CVD: Cardiovascular Disease
RCT: Randomised Controlled Trial
CPAP: Continuous Positive Airway Pressure
PA: Physical Activity
AHI: Apnoea/Hypopnoea Index
RPE: Rate of Perceived Exertion
REM: Rapid Eye Movement
IPAQ: International Physical Activity Questionnaire
BMI: Body Mass Index
ANOVA: Analysis Of Variance
SPSS: Statistical Package for the Social Sciences

## References

[1] Benjafield A, Valentine K, Ayas N, Eastwood PR, Heinzer RC, Patel M (2018). Global prevalence of obstructive sleep apnea in adults: estimation using currently available data. Am J Respir Crit Care Med Abstract.

[2] Punjabi NM, Caffo BS, Goodwin JL, Gottlieb DJ, Newman AB, O'Connor GT (2009). Sleep-disordered breathing and mortality: a prospective cohort study. PLoS Med.

[3] Al Lawati NM, Patel SR, Ayas NT (2009). Epidemiology, risk factors, and consequences of obstructive sleep apnea and short sleep duration. Prog Cardiovasc Dis.

[4] Punjabi NM (2008). The epidemiology of adult obstructive sleep apnea. Proc Am Thorac Soc.

[5] Rejon-Parrilla JC, Garau M, Sussex J. Obstructive sleep Apnoea health economics report. Office Health Econ. 2014:1–39.

[6] Tahrani AA (2017). Obstructive sleep apnoea in diabetes: does it matter?. Sage Publications.

[7] Floras JS (2014). Sleep apnea and cardiovascular risk. J Cardiol.

[8] Altaf QA, Dodson P, Ali A, Raymond NT, Wharton H, Fellows H (2017). Obstructive sleep apnea and retinopathy in patients with type 2 diabetes. A longitudinal study. Am J Respir Crit Care Med.

[9] Tahrani AA, Ali A, Raymond NT, Begum S, Dubb K, Mughal S (2012). Obstructive sleep apnea and diabetic neuropathy: a novel association in patients with type 2 diabetes. Am J Respir Crit Care Med.

[10] Pamidi S, Tasali E (2012). Obstructive sleep apnea and type 2 diabetes: is there a link?. Front Neurol.

[11] Tahrani AA, Ali A, Raymond NT, Begum S, Dubb K, Altaf QA (2013). Obstructive sleep apnea and diabetic nephropathy. Diabetes Care.

[12] Zhang W, Si L (2012). Obstructive sleep apnea syndrome (OSAS) and hypertension: pathogenic mechanisms and possible therapeutic approaches. Ups J Med Sci.

[13] Sawyer AM, Gooneratne NS, Marcus CL, Ofer D, Richards KC, Weaver TE (2011). A systematic review of CPAP adherence across age groups: clinical and empiric insights for developing CPAP adherence interventions. Sleep Med Rev.

[14] Iftikhar IH, Bittencourt L, Youngstedt SD, Ayas N, Cistulli P, Schwab R (2017). Comparative efficacy of CPAP, MADs, exercise-training, and dietary weight loss for sleep apnea: a network meta-analysis. Sleep Med.

[15] McEvoy RD, Antic NA, Heeley E, Luo Y, Ou Q, Zhang X (2016). CPAP for prevention of cardiovascular events in obstructive sleep apnea. N Engl J Med.

[16] Cvengros JA, Rodriguez VM, Snyder S, Hood MM, Crawford M, Park M. An adaptive treatment to improve positive airway pressure (PAP) adherence in patients with obstructive sleep apnea: a proof of concept trial. Behav Sleep Med. 2016:1–16. 10.1080/15402002.2015.1135292 27096396

[17] Tyrrell J, Poulet C, Pe Pin JL, Veale D (2006). A preliminary study of psychological factors affecting patients’ acceptance of CPAP therapy for sleep apnoea syndrome. Sleep Med.

[18] Yetkin O, Kunter E, Gunen H (2008). CPAP compliance in patients with obstructive sleep apnea syndrome. Sleep Breathing.

[19] Tan X, Alen M, Cheng SM, Mikkola TM, Tenhunen J, Lyytikainen A (2015). Associations of disordered sleep with body fat distribution, physical activity and diet among overweight middle-aged men. J Sleep Res.

[20] Simpson L, McArdle N, Eastwood PR, Ward KL, Cooper MN, Wilson AC (2015). Physical inactivity is associated with moderate-severe obstructive sleep apnea. J Clin Sleep Med.

[21] Verwimp J, Ameye L, Bruyneel M (2013). Correlation between sleep parameters, physical activity and quality of life in somnolent moderate to severe obstructive sleep apnea adult patients. Sleep Breathing.

[22] Peppard PE, Young T (2004). Exercise and sleep-disordered breathing: an association independent of body habitus. Sleep..

[23] Wahid A, Manek N, Nichols M, Kelly P, Foster C, Webster P, et al. Quantifying the Association Between Physical Activity and Cardiovascular Disease and Diabetes: A Systematic Review and Meta-Analysis. J Am Heart Assoc. 2016;5(9). 10.1161/JAHA.115.002495 PMC5079002 27628572

[24] Katzmarzyk PT, Church TS, Blair SN. Cardiorespiratory fitness attenuates the effects of the metabolic syndrome on all-cause and cardiovascular disease mortality in men. Arch Intern Med. 2004;164. 10.1001/archinte.164.10.1092 15159266

[25] Redolfi S, Bettinzoli M, Venturoli N, Ravanelli M, Pedroni L (2015). Attenuation of obstructive sleep apnea and overnight rostral fluid shift by physical activity. Am J Respir Crit Care Med.

[26] Vincent HK, Shanely RA, Stewart DJ, Demirel HA, Hamilton KL, Ray AD (2002). Adaptation of upper airway muscles to chronic endurance exercise. Am J Respir Crit Care Med.

[27] Browne RH (1995). On the use of a pilot sample for sample size determination. Stat Med.

[28] Crowley KE, Rajaratnam SM, Shea SA, Epstein LJ, Czeisler CA, Lockley SW (2013). Evaluation of a single-channel nasal pressure device to assess obstructive sleep apnea risk in laboratory and home environments. J Clin Sleep Med.

[29] Craig CL, Marshall AL, Sjostrom M, Bauman AE, Booth ML, Ainsworth BE (2003). International physical activity questionnaire: 12-country reliability and validity. Med Sci Sports Exerc.

[30] Santos-Lozano A, Marin PJ, Torres-Luque G, Ruiz JR, Lucia A, Garatachea N (2012). Technical variability of the GT3X accelerometer. Med Eng Phys.

[31] Cain KL, Geremia CM. Accelerometer Data Collection and Scoring Manual. For Adult & Senior Studies. 2011.

[32] Freedson PS, Melanson E, Sirard J (1998). Calibration of the computer science and applications, Inc. accelerometer. Med Sci Sports Exerc.

[33] Scherr J, Wolfarth B, Christle JW, Pressler A, Wagenpfeil S, Halle M (2013). Associations between Borg's rating of perceived exertion and physiological measures of exercise intensity. Eur J Appl Physiol.

[34] Kushner RF, Kunigk A, Alspaugh M, Andronis PT, Leitch CA, Schoeller DA (1990). Validation of bioelectrical-impedance analysis as a measurement of change in body composition in obesity. Am J Clin Nutr.

[35] Clark AL, Crabbe S, Aziz A, Reddy P, Greenstone M (2009). Use of a screening tool for detection of sleep-disordered breathing. J Laryngol Otol.

[36] Patel MR, Alexander TH, Davidson TM (2007). Home sleep testing. Oper Tech Otolaryngol Head Neck Surg.

[37] AASM (1999). Task Force. Sleep-related breathing disorders in adults: recommendations for syndrome definition and measurement techniques in clinical research. Rep Am Acad Sleep Med Task Force Sleep.

[38] Chowdhuri S, Quan SF, Almeida F, Ayappa I, Batool-Anwar S, Budhiraja R (2016). An official American Thoracic Society research statement: impact of mild obstructive sleep apnea in adults. Am J Respir Crit Care Med.

[39] American Academy of Sleep Medicine. The AASM Manual for the Scoring of Sleep and Asociated Events Version 2.4. 2017.

[40] Ucok K, Aycicek A, Sezer M, Genc A, Akkaya M, Caglar V (2009). Aerobic and anaerobic exercise capacities in obstructive sleep apnea and associations with subcutaneous fat distributions. Lung..

[41] Sengul YS, Ozalevli S, Oztura I, Itil O, Baklan B (2011). The effect of exercise on obstructive sleep apnea: a randomized and controlled trial. Sleep Breathing.

[42] Alves Eda S, Ackel-D'Elia C, Luz GP, Cunha TC, Carneiro G, Tufik S (2013). Does physical exercise reduce excessive daytime sleepiness by improving inflammatory profiles in obstructive sleep apnea patients?. Sleep Breathing.

[43] de Andrade FM, Pedrosa RP (2016). The role of physical exercise in obstructive sleep apnea. J Bras Pneumol.

[44] Iftikhar IH, Kline CE, Youngstedt SD (2014). Effects of exercise training on sleep apnea: a meta-analysis. Lung..

[45] Kline CE, Crowley EP, Ewing GB, Burch JB, Blair SN, Durstine JL (2011). The effect of exercise training on obstructive sleep apnea and sleep quality: a randomized controlled trial. Sleep..

[46] Chaput JP, Carson V, Gray CE, Tremblay MS (2014). Importance of all movement behaviors in a 24 hour period for overall health. Int J Environ Res Public Health.

[47] Fleg JL, Strait J (2012). Age-associated changes in cardiovascular structure and function: a fertile milieu for future disease. Heart Fail Rev.

[48] Leino-Arjas P, Solovieva S, Riihimaki H, Kirjonen J, Telama R (2004). Leisure time physical activity and strenuousness of work as predictors of physical functioning: a 28 year follow up of a cohort of industrial employees. Occup Environ Med.

[49] Ejaz SM, Khawaja IS, Bhatia S, Hurwitz TD (2011). Obstructive sleep apnea and depression: a review. Innov Clin Neurosci.

[50] Thomasouli MA, Brady EM, Davies MJ, Hall AP, Khunti K, Morris DH (2013). The impact of diet and lifestyle management strategies for obstructive slee apnoea in adults: a systematic review and meta-analysis of randomised controlled trials. Sleep Breathing.

[51] Giebelhaus V, Strohl KP, Lormes W, Lehmann M, Netzer N (2000). Physical exercise as an adjunct therapy in sleep apnea - an open trial. Sleep Breathing.

[52] Aiello KD, Caughey WG, Nelluri B, Sharma A, Mookadam F, Mookadam M (2016). Effect of exercise training on sleep apnea: a systematic review and meta- analysis. Respir Med.

[53] Eckel RH, Jakicic JM, Ard JD, de Jesus JM, Houston Miller N, Hubbard VS, et al. 2013 AHA/ACC guideline on lifestyle management to reduce cardiovascular risk: a report of the American College of Cardiology/American Heart Association task force on practice guidelines. J Am Coll Cardiol. 2014;63(25 Pt B):2960-2984. 10.1016/j.jacc.2013.11.003 24239922

[54] Spencer RM, Heidecker B, Ganz P (2016). Behavioral cardiovascular risk factors- effect of physical activity and cardiorespiratory fitness on cardiovascular outcomes. Circ J.

[55] Mendelson M, Bailly S, Marillier M, Flore P, Borel JC, Vivodtzev I, et al. Obstructive sleep apnea syndrome, objectively measured physical activity and exercise training interventions: a systematic review and meta-analysis. Front Neurol. 2018;9:73. 10.3389/fneur.2018.00073 PMC5827163 29520251

